# The Effect of Coil Orientation on the Stimulation of the Pre–Supplementary Motor Area: A Combined TMS and EEG Study

**DOI:** 10.3390/brainsci12101358

**Published:** 2022-10-06

**Authors:** Elias P. Casula, Giorgio Leodori, Jaime Ibáñez, Alberto Benussi, Vishal Rawji, Sara Tremblay, Anna Latorre, John C. Rothwell, Lorenzo Rocchi

**Affiliations:** 1 Non−Invasive Brain Stimulation Unit, IRCCS Santa Lucia Foundation, 00179 Rome, Italy; 2 Department of Clinical and Movement Neurosciences, UCL Queen Square Institute of Neurology, University College London, London WC1N 3BG, UK; 3 Department of Human Neurosciences, Sapienza University of Rome, 00185 Rome, Italy; 4 IRCCS Neuromed, 86077 Pozzilli, Italy; 5 Department of Bioengineering, Imperial College, London SW7 2AZ, UK; 6 BSICoS Group, I3A Institute, University of Zaragoza, IIS Aragón, 50009 Zaragoza, Spain; 7 Neurology Unit, Department of Clinical and Experimental Sciences, University of Brescia, 25121 Brescia, Italy; 8 The Royal’s Institute of Mental Health Research, University of Ottawa, Ottawa, ON K1N 6N5, Canada; 9 Département de Psychoéducation et Psychologie, Université du Québec en Outaouais, Gatineau, QC J8X 3X7, Canada; 10 Department of Cellular and Molecular Medicine, Faculty of Medicine, University of Ottawa, Ottawa, ON K1N 6N5, Canada; 11 Department of Medical Sciences and Public Health, University of Cagliari, 09124 Cagliari, Italy

**Keywords:** transcranial magnetic stimulation, electroencephalography, motor evoked potentials, TMS–EEG, presupplementary motor area, coil direction

## Abstract

Studies using transcranial magnetic stimulation (TMS) have demonstrated the importance of direction and intensity of the applied current when the primary motor cortex (M1) is targeted. By varying these, it is possible to stimulate different subsets of neural elements, as demonstrated by modulation of motor evoked potentials (MEPs) and motor behaviour. The latter involves premotor areas as well, and among them, the presupplementary motor area (pre–SMA) has recently received significant attention in the study of motor inhibition. It is possible that, similar to M1, different neuronal populations can be activated by varying the direction and intensity of TMS; however, the absence of a direct electrophysiological outcome has limited this investigation. The problem can be solved by quantifying direct cortical responses by means of combined TMS and electroencephalography (TMS–EEG). We investigated the effect of variable coil orientations (0°, 90°, 180° and 270°) and stimulation intensities (100%, 120% and 140% of resting motor threshold) on local mean field potential (LMFP), transcranial evoked potential (TEP) peaks and TMS–related spectral perturbation (TRSP) from pre–SMA stimulation. As a result, early and late LMFP and peaks were larger, with the coil handle pointing posteriorly (0°) and laterally (90°). This was true also for TRSP in the β–γ range, but, surprisingly, θ–α TRSP was larger with the coil pointing at 180°. A 90° orientation activated the right M1, as shown by MEPs elicitation, thus limiting the spatial specificity of the stimulation. These results suggest that coil orientation and stimulation intensity are critical when stimulating the pre–SMA.

## 1. Introduction

Transcranial magnetic stimulation (TMS) is a noninvasive brain stimulation technique that has been extensively used in the past decades to study the basic physiology of the cerebral cortex, as well as human behaviour in health and disease. Most studies so far have been performed on the primary motor area (M1); a single TMS pulse applied here activates excitatory inputs to corticospinal neurons. This ultimately leads to the generation of descending volleys, which give rise to a compound muscle action potential called motor evoked potential (MEP) [1]. The current direction induced in the brain by the TMS pulse has a critical role in this process. It is well known that the latency of the MEP is longer if the current applied has an anterior to posterior (AP) direction compared with posterior to anterior (PA). Possibly, this is due to the AP and PA currents activating different sets of inputs to corticospinal neurons [2,3,4,5,6,7]. Besides coil orientation (CO), this differential activation also depends on the stimulation intensity (SI) used, i.e., a low SI is more selective in activating subsets of inputs to corticospinal neurons [5]. Even more importantly, this specific activation seems to be behaviourally relevant: it has been suggested that the suppression of a set of motor cortical neurons, obtained by a specific current direction of TMS, may have an impact on movement preparation [8,9].

Motor planning is a complex phenomenon also involving higher–order motor areas, including the presupplementary motor area (pre–SMA). In humans, it is located in the dorsomedial frontal cortex, anterior to the leg representation of the primary motor cortex (M1) [10,11]. Several lines of evidence have pointed towards a role of the pre–SMA in complex motor behaviour, including self–initiated activity, generation of action sequences and motor learning [12]. This has been confirmed in human studies using TMS; in particular, the human pre–SMA has received attention in the context of switching and stopping behaviour [13,14,15,16,17]. However, in most studies, parameters such as CO and SI have been selected without a clear rationale. It is possible that, as for M1, different neuronal populations can be activated in the pre–SMA by varying CO and SI; however, investigating this outside M1 is difficult due to the absence of a direct readout such as MEP. This problem can be solved with the use of combined TMS and electroencephalography (TMS–EEG), which allows to record postsynaptic potentials generated by the magnetic pulse in the form of transcranial evoked potentials (TEPs) [7,18,19,20] or oscillations [21,22], and, thus, to have a readout from cortical areas outside M1.

In the present study, we assessed TMS–evoked EEG responses from the pre–SMA in a cohort of young, healthy individuals, with the aim of investigating signal changes due to different COs and SIs. To assess cortical excitability, we analysed TEPs in terms of discrete peaks and local mean field potential (LMFP), a reference–free measure commonly used to measure local excitability of a specific area, from a cluster of nearby electrodes [6,23,24]. To assess cortical oscillations, we computed TMS–related spectral perturbation (TRSP), a measure reflecting the power of TMS–evoked response in the frequency domain [21,22,24]. These variables were computed locally to the SMA since we were interested in the response of this area obtained with three different SIs (100%, 120% and 140%) of resting motor threshold (RMT) and four different COs (0°, 90°, 180° and 270°, starting with the coil handle pointing posteriorly on the transverse plane and proceeding counterclockwise). Overall, we found that both CO and SI are critical in determining TMS–EEG responses, and this likely suggests that, by varying these parameters, it is possible to preferentially stimulate different neural elements within the pre–SMA.

## 2. Materials and Methods

### 2.1. Subjects and Experimental Sessions

**Figure 1:**
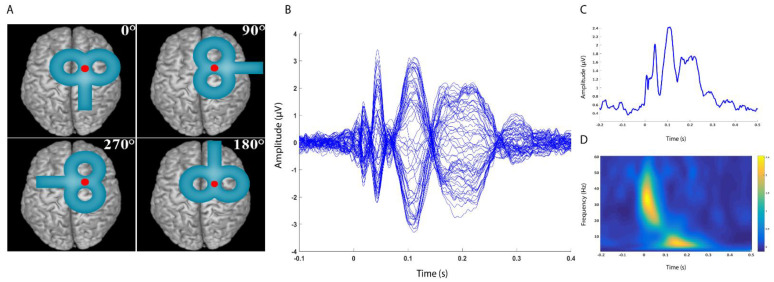
Panel (**A**): Experimental protocol. Single–pulse TMS was applied on the right pre–SMA using four different coil orientations (0°, 90°, 180°, 270°), starting with the coil handle pointing posteriorly on the transverse plane and proceeding counterclockwise). For each coil orientation, three stimulation intensities were used (100%, 120% and 140% of the RMT). Panel (**B**): Example of TEP obtained by averaging signals from all subjects in the 0° CO and 140% SI condition. Each line represents a signal from one electrode; all 62 recording electrodes are plotted. Panel (**C**): Example of LMFP obtained by averaging signals from all subjects in the 0° CO and 140% SI condition. Panel (**D**): Example of TRSP obtained by averaging signals from all subjects in the 0° CO and 140% SI condition.

Sixteen healthy subjects (7 female, age 29.5 ± 4.6), all right–handed [25], were enrolled in the study. They had no history of neurological or psychiatric diseases and were not taking drugs active at the central nervous system level at the time of the experiment. Subjects gave their written consent to participate prior to the experimental sessions. All procedures were performed in accordance with the Declaration of Helsinki. Approval to conduct the experiments was obtained from the human subjects review board of the University College London. The experiment consisted of two sessions; participants underwent a total of 12 blocks of TMS–EEG recording, in which four different COs (0°, 90°, 180° and 270°, starting with the coil handle pointing posteriorly on the transverse plane and proceeding counterclockwise) and, for each of them, three different SIs (100%, 120% and 140% RMT) were tested (Figure 1, panel A). Each recording block consisted of 100 TMS pulses, and the order of the blocks was randomised across the two sessions and within the same session. Single–pulse TMS was applied over the right pre–SMA during EEG recording with an interpulse interval of 4 ± 10% s (3.6–4.4 s); EMG was recorded bilaterally from the first dorsal interosseous (FDI) muscle to check whether stimulation was able to induce MEPs.

### 2.2. Electric Field Modelling

To ensure that the chosen stimulation intensities could generate an electric field (E–field) in the cortex sufficient for a reliable TEP, i.e., at least 40 V/m [22,26], we computed the induced E–field over the TMS target with SimNIBS v3.2, an open–source simulation package that integrates segmentation of MRI scans, mesh generation and FEM E–field estimate [27]. The E–field was computed for the twelve stimulation conditions resulting from the combination of four COs (0°, 90°, 180°, 360°) and three SIs (100%, 120%, 140% RMT). The E–fields were estimated based on the MNI standard brain (ERNIE) provided in SimNIBS software as an anatomical reference [28].

### 2.3. TMS, Electromyographic Recording and Analysis

EMG activity was recorded through a pair of Ag/AgCl electrodes placed over the right and left FDI muscle in a belly–tendon montage. EMG signal was amplified and filtered (gain 1000x; bandwidth 5 Hz–2 kHz) with a Digitimer D360 (Digitimer Ltd., Welwyn Garden City, UK), then digitally converted with a CED 1401 analogue–to–digital laboratory interface (Cambridge Electronic Design Ltd., Milton, Cambridge, UK). Single–pulse TMS was performed using a Magstim 200 stimulator with a 70 mm figure of eight coil (Magstim Co Ltd., Whitland, UK), which produces stimuli with a monophasic waveform and a pulse width of ~80 µs.

The RMT was measured on the FDI hotspot of the right M1, which was defined as the site where TMS evoked the largest MEP in the left FDI muscle. The RMT was calculated as the lowest magnetic stimulator intensity able to evoke an MEP of at least 50 µV in 5 out of 10 consecutive trials in the relaxed FDI [29,30]. Peak–to–peak amplitudes of MEP were calculated by summing the absolute minimum and maximum EMG values in a region between 10 and 60 ms after the TMS artefact. The right pre–SMA cortical site where TMS was applied was identified based on MNI coordinates from previous studies (x = 20; y = 6; z = 62) [31]. These coordinates were used to initially locate the area and to maintain the coil in the correct position throughout the stimulation blocks by using a Brainsight navigation system (Brainbox Ltd., Cardiff, UK) coupled with a Polaris Spectra optical measurement system (Northern Digital Inc, Waterloo, Canada). The chosen location is more lateral and posterior compared with the pre–SMA identified elsewhere [32], and it has been specifically linked to correct stopping behaviour during a stop signal task [31]. Note that this area is far from craniofacial muscles, whose activation by TMS could potentially affect the EEG signal [23,33]. An estimated individualised MRI scan in the MNI space was used for all the participants. Previous studies demonstrated that the mean accuracy of the estimated MRI scans is comparable with the spatial resolution of TMS [34]. To increase the reproducibility of our data between the two sessions, in the first experiment, the position of the recording electrodes was digitised in the MNI space, and the coordinates were used to ensure their accurate placement in the second experiment.

### 2.4. Electroencephalographic Recording and Analysis

EEG was recorded using a TMS–compatible amplifier (TruScan EEG, Deymed Diagnostic s.r.o, Hronov, Czech Republic). The system minimises the TMS–induced artefact by removing the AC coupling from 0.5 ms before to 1 ms after the TMS pulse. Signals were recorded from 62 TMS–compatible Ag/AgCl passive electrodes mounted on a cap produced by the same manufacturer, according to the 10–20 international EEG system, including: Fp1, Fp2, F7, F3, Fz, F4, F8, T7, C3, Cz, C4, T8, P7, P3, Pz, P4, P8, O1, O2, FC5, FC1, FC2, FC6, AF7, CP5, CP1, CP2, CP6, AF8, Oz, FPz, AF3, FC3, AF4, C6, Iz, FC4, FT8, F5, C2, F1, AFz, C5, F2, TP7, F6, C1, FCz, FT7, CP3, CPz, CP4, TP8, P5, P1, P2, P6, PO7, PO3, POz, PO4 and PO8.

Recordings were referenced online to linked mastoids, and the ground electrode was placed above the nasion. Skin impedances were kept below 5 kΩ, and the sampling frequency during recording was 3000 Hz. In order to mask the TMS–induced noise and avoid possible auditory evoked potentials, participants wore earplugs continuously playing a white noise mixed with specific time–varying frequencies of the TMS [35,36]. Additionally, a 0.5 cm foam layer was placed underneath the coil to minimise bone conduction of the TMS click and scalp sensation caused by coil vibration.

Offline EEG preprocessing was performed with EEGLAB 14.1.1 [37] with the addition of some functions included in the TMS−EEG signal analyser (TESA) toolbox [38] and in Fieldtrip open−source MATLAB toolbox [39], all running in MATLAB environment (Version 2016b, MathWorks Inc., Natick, MA, USA).

EEG signal recorded in all blocks was epoched (−1.3 to 1.3 s) and demeaned using a baseline from −1000 to −10 ms. Epochs were visually inspected, and those with excessively noisy EEG were excluded. The TMS artefact was removed from −5 to 10 ms around the trigger and interpolated by means of a cubic function. A first round of independent component decomposition analysis (ICA) was run using a fastICA algorithm. Only the 15 components explaining the largest variance were plotted in a time window ranging from −200 to 500 ms, and those reflecting residual scalp muscle or voltage decay artefacts were eliminated after visual inspection based on time, frequency, scalp distribution and amplitude criteria [40,41]. After this, a band–pass (1–100 Hz) and a band–stop (48–52 Hz) fourth−order Butterworth filter were applied. The signal was further epoched (−1 to 1 s) to reduce possible edge artefacts, and a second round of fastICA was performed to remove residual artefacts (e.g., eyeblinks, continuous muscle activity, etc.). Lastly, a common average reference was applied.

**Figure 2:**
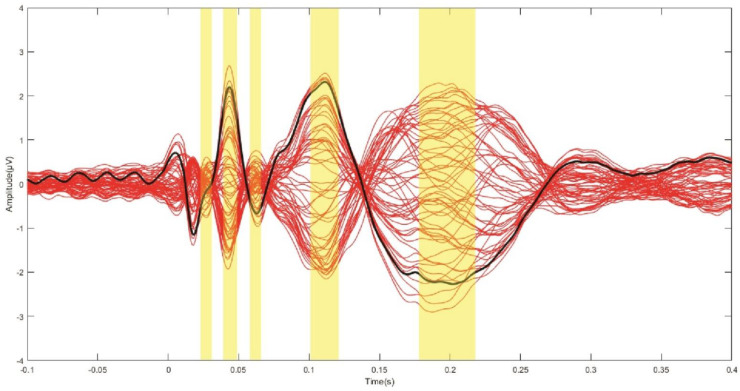
Grande average TEP across subjects and conditions. Each red line depicts the TEP from one electrode. The thick black line indicates the TEP averaged across the four electrodes from which the LMFP was calculated (Fz, F2, FCz, FC2). The yellow panels indicate the time windows from which maximum/minimum values of TEP peak amplitudes were extracted (see text for details).

Since the aim of the study was to investigate the local effects related to the variation in CO and SI, we calculated several TMS–EEG measures in a cluster of electrodes surrounding the stimulation site (Fz, F2, FCz, FC2). To assess the local cortical activation induced by TMS in the time domain, we computed the LMFP as the square root of squared TEPs averaged across the four channels of interest, as performed in previous studies [23,24,42,43]. The time–domain signal was further analysed, considering discrete TEP peaks. After computing the grand average signal across all subjects and conditions, we identified five main waves by visual inspection, peaking at 26 (PI), 44 (PII), 62 (PIII), 118 (PIV) and 198 (PV) ms. Maximum (for positive) and minimum (for negative) amplitude values were extracted for each subject, condition and peak within the following time windows: 22–30 ms (PI), 39–49 ms (PII), 58–66 ms (PIII), 108–128 ms (PIV) and 178–228 ms (PV) (Figure 2).

For the time–frequency analysis of TEP, spectral estimations of the EEG epochs were obtained for frequencies between 1 and 60 Hz (1 Hz resolution) and times in the interval from −500 to 500 ms. A sliding window (5 ms steps), linearly increasing its length across frequencies (1 cycle length for 1 Hz up to 7 cycles for 60 Hz), was used to extract amplitude and power values of all time–frequency bins. These values were estimated using the multitapers method as implemented in fieldtrip’s ft_freqanalysis function. For these estimations, Hanning tapers were used, and the amount of spectral smoothing factor was set to 0.1 times the frequency analysed in each bin. Then, TRSP was computed as follows:

$$TRSP\left(f,t\right)=\frac{1}{n}\overset{n}{\underset{k=1}{\sum }}{\left|F_{k}\left(f,t\right)\right|}^{2}$$

where, for *n* trials, the spectral power estimate *F* was computed at trial *k*, at frequency *f* and time *t* [37]. Both the LMFP and the TRSP were measured in two time windows (early: 10–70 ms; late: 70–250 ms); these should reflect more local vs. more distributed brain activation, respectively [36]. Another rationale for the indicated time windows was to compare the LMFP and TRSP, the latter showing separate response clusters in the two intervals (Figure 1, panels C and D). TSRP values were averaged from 10 to 70 ms for γ (31–48 Hz) and β (14–30 Hz) frequency bands and from 70 to 250 ms for α (8–13 Hz) and θ (5–8 Hz) frequency bands.

### 2.5. Statistical Analysis

To measure differences in LMFP induced by TMS delivered with different CO and SI, we performed two separate two–way repeated measures ANOVAs on early and late LMFP, respectively. Factors of analysis were “CO” (0°, 90°, 180° and 270°) and “SI” (100%, 120% and 140% RMT). Five ANOVAs with the same structure were used to assess the effects of CO and SI on TEP peaks. Possible differences in TRSP were assessed again by means of repeated measures ANOVAs with the same factors and levels as before. This time, values were averaged both across frequencies and time windows (10–70 ms for β–γ range and 70–250 ms for θ–α range). To check whether TMS over the pre−SMA effectively stimulated M1, we also performed a three–way ANOVA on MEP amplitude, using “side” (left, right) “SI” (100%, 120% and 140% RMT) and “CO” (0°, 90°, 180° and 270°) as factors of analysis. Before undergoing ANOVAs, normal distribution of data was assessed by means of Shapiro–Wilk’s test. All *p*-values < 0.05 were considered significant. Greenhouse–Geisser correction was used when necessary to correct for non–sphericity (i.e., Mauchly’s test < 0.05). To correct for multiple comparisons, Bonferroni’s correction was used for main effects, interactions and post hoc analyses following the ANOVAs. Statistical analyses were performed with IBM SPSS v24 (Armonk, NY, USA: IBM Corp).

## 3. Results

The test sessions were well tolerated, and no participants reported any side effects. Results are expressed as average ± standard deviation (SD) if not otherwise specified. Average RMT was 55.1 ± 9.4 of the maximum stimulator output (MSO). TMS pulses induced a pattern of negative and positive deflections consistent with previous literature [44,45] (Figure 1, panel B; Figure 2). The TRSP showed a prominent and early increase in power peaking in the γ frequency range, compatible with activation of medial motor areas [26], and a later increase in the θ–α frequency bands (Figure 1, panel D).

### 3.1. Electric Field Modelling

**Figure 3:**
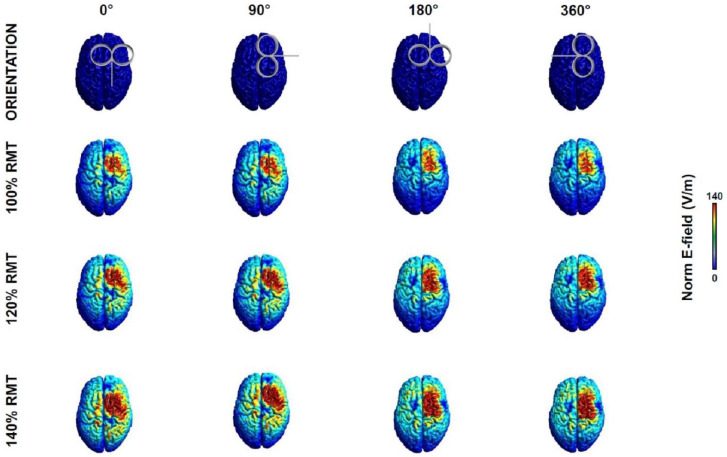
Pictorial representation of the E–field induced by TMS in the 12 conditions explored (see text for details).

Table 1 and Figure 3 report the E–fields computed for the 12 conditions explored. Our results showed that, even with the lowest intensity of stimulation (100% RMT), the estimated E–fields were well above the threshold of 40 V/m to evoke a reliable TMS–evoked EEG response [22,26]. To note that the E–fields obtained at 0° and 90° CO were higher than those at 180° and 270°; this might, to an extent, have contributed to our results (see below).

### 3.2. Local Mean Field Potential and TEP Peaks

**Figure 4:**
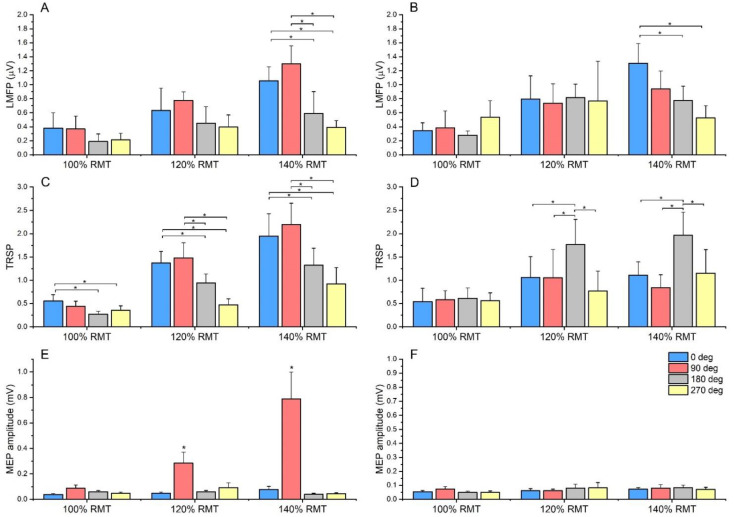
Summary of results on LMFP and TRSP. Panel (**A**): Early LMFP (10–70 ms) was larger at 0° and 90° compared with 180° and 270°. Although this was true for all the tested SIs, the effect reached statistical significance only at 140% RMT. Panel (**B**): Late LMFP (70–250 ms) evoked by 0° CO was larger than 180° and 270°, and again, this occurred only when an SI of 140% RMT was used. Panel (**C**): For TRSP in the β–γ frequency range, 0° CO induced a higher TRSP than 180° and 270° at 100% SI. At higher intensities, both 0° and 90° COs induced a higher TRSP compared with 180° and 270°. Panel (**D**): TRSP in the θ–α frequency range showed higher values for 180° compared with the other CO, both at 120% and 140% RMT SIs. Panel (**E**): MEP recorded from the left FDI were larger with 90° CO when using 120% and 140% RMT SIs compared with all other conditions. Panel (**F**): MEP recorded from the right FDI showed no difference across different CO and SI. Note: TRSP is expressed as the relative change compared with baseline. Error bars indicate the standard error of the mean. Asterisks indicate statistical significance (* *p* < 0.05).

Globally, LMFP induced by the 0° and 90° CO was larger than 180° and 270°. The ANOVA on early LMFP showed a significant main effect of “CO” (F_3,39_ = 5.736, *p* = 0.02), “SI” (F_2,26_ = 9.731, *p* < 0.001) and a significant “CO × SI” interaction (F_6,78_ = 3.12, *p* = 0.04). Post hoc comparisons showed a consistently larger LMFP at 0° and 90° CO compared with 180° and 270°. Although this was true for all the tested SIs, the effect reached statistical significance only at 140% RMT (all *p* values < 0.01). A similar pattern was found in the ANOVA on late LMFP, where a significant main effect of “CO” (F_3,39_ = 3.506, *p* = 0.024), “SI” (F_2,26_ = 11.804, *p* < 0.001) and a significant “CO × SI” interaction (F_6,78_ = 4.633, *p* < 0.001) were found. This time, only LMFP evoked by 0° was larger than 180° and 270°, and this again occurred only when an SI of 140% RMT was used (Figure 4).

**Figure 5:**
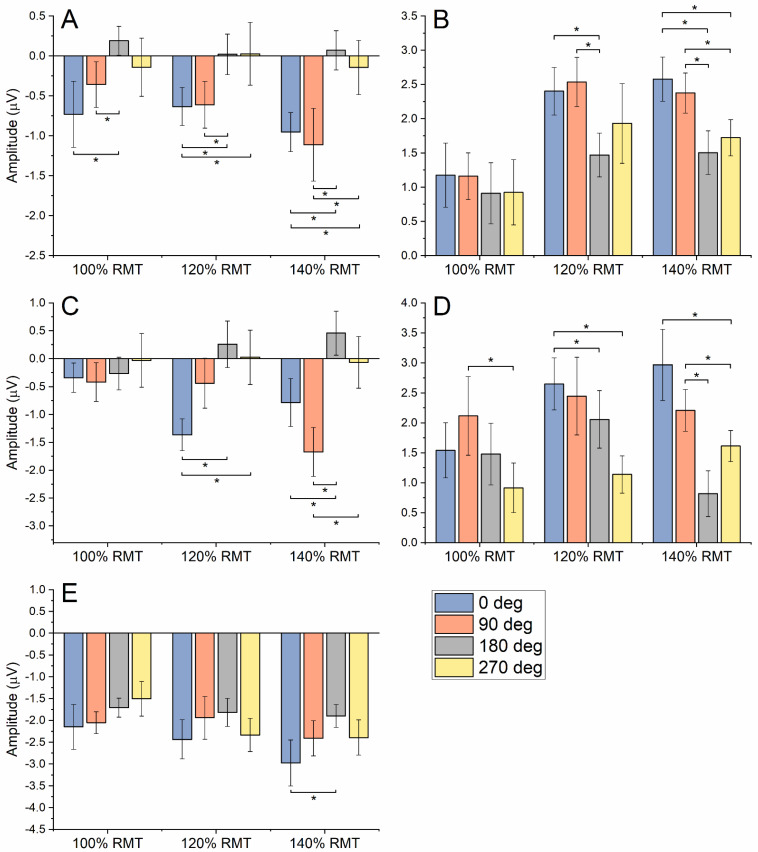
Summary of results on TEP peaks: panel (**A**), PI; panel (**B**), PII; panel (**C**)**,** PIII; panel (**D**), PIV; panel (**E**), PV. Error bars indicate standard error of the mean. Asterisks indicate statistical significance (* *p* < 0.05).

The results of the ANOVAs on TEP peaks are summarised in Table 2 and Figure 5. Overall, these analyses confirmed the trend observed for the LMFP, i.e., larger amplitude values for 0° and 90° CO compared with 180° and 270°. Again, this was clearer with increasing stimulation intensities, except for PI and PIV, in which some statistically significant differences were observed for 100% RMT as well (Figure 5).

### 3.3. TMS–Related Spectral Perturbation

TRSP showed a trend similar to LMFP and TEP peaks in the early time window, in which power in the β–γ bands was considered. The ANOVA on TRSP at β–γ frequencies showed a significant main effect of “CO” (F_3,39_ = 8.144, *p* < 0.001), “SI” (F_3,39_ = 53.502, *p* < 0.001) and a significant “CO × SI” interaction (F_3,39_ = 3.816, *p* = 0.02). Post hoc comparisons showed that 0° induced a higher TRSP than 180° and 270° at 100% SI (*p* = 0.023 and 0.015, respectively). At higher intensities (120% and 140% RMT), both 0° and 90° COs induced a higher TRSP compared with 180° and 270° (all *p* values < 0.01). Interestingly, results were very different in the TRSP measured in the θ–α bands. In this case, the ANOVA showed a significant main effect of “CO” (F_3,39_ = 7.467, *p* = 0.01), “SI” (F_3,39_ = 27.433, *p* < 0.001) and a significant “CO × SI” interaction (F_3,39_ = 5.318, *p* < 0.001). This time, a higher TRSP was induced by stimulation at 180° compared with the other three COs, and this was true for 120% and 140% RMT SIs (all *p* values < 0.01) (Figure 4).

### 3.4. Motor Evoked Potentials

No stimulation condition elicited MEPs in the right FDI, whereas clear MEPs were recorded in the left FDI with a 90° CO, both at 120% and 140% SIs. The related ANOVA showed a significant main effect of “side” (F_1,15_ = 10.309, *p* = 0.006), “CO” (F_3,45_ = 11.947, *p* < 0.001), “SI” (F_2,30_ = 10.414, *p* < 0.001), as well as significant interactions of “side × CO” (F_3,45_ = 11.579, *p* < 0.001), “side × SI” (F_2,30_ = 7.979, *p* = 0.002), “CO × SI” (F_2,30_ = 10.474, *p* < 0.001) and “side × CO × SI” (F_2,30_ = 9.129, *p* < 0.001). Post hoc comparisons demonstrated that, when using 90° CO, both at 120% and 140% SIs, MEP size was larger compared with all other conditions (all *p* values < 0.05) (Figure 4).

## 4. Discussion

In this study, we demonstrated that TMS coil direction, already known to influence responses from M1, also plays a major role when the pre–SMA is stimulated. Specifically, early time–domain, late time–domain, and early time/frequency–domain EEG responses to TMS were generally larger when the coil handle was pointing posteriorly and 90° laterally. Albeit this result might be partly explained by a larger E–field induced in these two conditions (Figure 3), it is likely that local neuronal dynamics contributed as well, since late TRSP was greater when the coil was pointing at 180°. Additionally, the present results suggest that stimulating the pre–SMA with a 90° orientation activates the right M1, thus limiting the spatial specificity of the stimulation.

Despite a large number of studies, TMS CO has not been sufficiently addressed when targeting the pre–SMA, both with single–pulse and repetitive TMS. So far, different TMS studies have used lateral [16,17] or posterior [15,46,47,48] CO. In some cases, CO was not specified [13,14], and in others, a cone coil was used [49,50,51]. Several studies specifically addressed the issue of TMS–EEG responses linked to different COs when M1 was stimulated. Results were mixed, with at least one reporting differences in TEP peaks [52]. The LMFP has been reported to be less sensitive to coil orientation, provided that stimulation intensity is adjusted by RMT when M1 is stimulated [6,7]. The effects of CO in other brain areas have been less studied. Casarotto and colleagues, for example [33], targeted a cortical spot in Brodmann’s area 6, possibly within the SMA; they found that CO affects EEG responses induced by TMS. However, in this study, as well as others investigating medial premotor areas [53,54], a biphasic stimulator was used, thus limiting inferences about the effects of CO on TEP.

The first important finding of the present study was that the early LMFP and corresponding TEP peaks (I–III), calculated between 10 and 70 ms after the TMS pulse, are larger with COs of 0° and 90° compared with 180° and 270°. Statistical significance was reached only at the maximal SI used (140% RMT) for the LMFP, while it also occurred for lower stimulation intensities for some peaks. This is in apparent contrast with previous investigations in M1, in which differences in MEP latency and modulation were obtained with a low stimulation intensity [5,55]. In this regard, it is worth noting that neurons in the pre–SMA have a higher threshold for electrical stimulation than those in M1 [56]. Additionally, they vary considerably in terms of sensitivity to somatosensory inputs and threshold to evoke movements [57]. It is thus possible that a higher TMS intensity recruits neurons with a higher threshold. Alternatively, since the spatial spread of the TMS–induced electric field depends on stimulation intensity, activation of cortical sites adjacent to the stimulated one may have contributed to CO–specific effects at high stimulation intensities. In this regard, since early TEPs mostly reflect local cortical activation [36], the spread of electric field within the same area may result in larger TEPs via in−phase summation of homogenous neural activity, whereas concurrent activation of different areas may have resulted in smaller TEPs via out–of–phase cancellation.

Interestingly, the observed effect was clearer on the β–γ TRSP compared with the LMFP. TRSP measured with a 0° CO was higher than 180° and 270° at all SIs. Similarly, TRSP at 90° was higher than 180° and 270° when a SI of 120% and 140% RMT were used. A possible explanation is that the response of the stimulated neurons is only partly phase–locked; thus, a measure such as the TRSP, which also takes into account nonphase–locked activity, is likely to be more sensitive to variations in the effectiveness of TMS. Such a notion has already been suggested to explain a higher sensitivity of TRSP compared with LMFP in detecting cortical excitability changes induced by continuous theta–burst stimulation [24]. The exact nature of the neural elements stimulated in the present study is difficult to ascertain. Previous data suggested that early components of TEP, which have frequency content in the beta range, could be a reflection of either excitatory postsynaptic potentials mediated by NMDA receptors [58] or inhibitory postsynaptic potentials due to GABAa receptors activation [59], the two hypotheses being not mutually exclusive.

When looking at longer latency responses, LMFP and TEP peaks showed a pattern similar to the earlier potentials. In this case, only 0° CO gave rise to significantly larger LMFP than 180° and 270°, and only at 140% RMT SI; by contrast, TEP PIV was modulated by CO even with lower stimulation intensities. Surprisingly, the pattern of θ–α TRSP was very different, i.e., it was larger with a CO of 180° compared with all the other COs, and the effect was significant at 120% and 140% RMT. It is thus possible that a CO of 180° stimulates neural elements which give rise to a more desynchronised global response; this might explain the discrepancy between time–domain signals and TRSP. Again, we can only speculate about the cellular mechanisms involved in these late responses to TEP. Some lines of evidence suggest that they might be mostly generated by local or interhemispheric inhibitory circuits involving GABAb receptors [60].

A further comment is needed with regards to the comparison between 0° and 90° COs. Albeit with slight differences in terms of post hoc comparisons with other COs, both gave rise to similar LMFP, TEP peaks and TRSP. However, a CO of 90° also evoked clear MEPs in the contralateral but not the ipsilateral FDI when SIs of 120% and 140% RMT were used. The most likely explanation is that when the coil handle points laterally, the magnetic field generated stimulates M1, which is slightly lateral to the pre–SMA. If this is the case, part of the signals observed when using a CO of 90° might be due to activation of M1, and thus it might be overestimated. Even if the measured cortical signal was not contaminated by activity generated in M1, the latter is nonetheless activated as indicated by MEP generation; thus, a CO of 90° might not be selective enough for stimulation of the pre–SMA. Lastly, part of the TMS–EEG signals observed in this case may be due to reafferent activity due to muscle twitch caused by MEP [43,61]. M1 activation might be partly dependent on the pre–SMA coordinates used here. We chose a site based on the peak of fMRI activation found by Sharp and colleagues in the context of motor response inhibition [31]. Compared to pre–SMA coordinates used in other studies [14], this site is more posterior and lateral, close to the border of the superior frontal gyrus and, thus, closer to M1; this might have facilitated M1 activation during our experiments.

It has recently been proposed that part of the TEP might be due to non–neural sources, including auditory and somatosensory evoked potentials, the latter being generated by activation of craniofacial muscles and cutaneous nerve fibres under the area of TMS stimulation [62,63,64]. However, as noted in the experimental procedure section, we took great care in minimising the contribution of non–neural sources by masking the TMS click with an appropriate noise and by placing a thin foam layer underneath the coil. It is also worth noticing that, due to the stimulated area being medial, scalp muscle activation was minimised [23,33] and that the remaining sources of the EEG signal not caused by direct cortical activation (e.g., afferent volleys generated by activation of somatosensory fibres in the skin) would not differ across experimental blocks. Additionally, since we compared conditions that were homogeneous in terms of stimulation intensity and scalp position, we are confident that our conclusion applies even if part of the responses were due to sensory input.

## 5. Conclusions

To sum up, the present findings suggest that, by varying CO and SI, it might be possible to target different neural populations in the pre–SMA, with different properties in terms of stimulation threshold and response synchronisation to the TMS pulse. It is known that neurons within the pre–SMA subserve different functions. These include modality–specific changes of activity in the context of reaching movements [65] and encoding information for the numerical order of components in sequences of movements [57,66]. Additionally, some neurons in the pre–SMA might be involved in non–motor tasks, such as comprehension of accelerated speech [67], and they may show variable sensitivity to somatosensory stimuli and threshold to evoke movements [57]. This evidence considered, further research is warranted to understand whether the different TMS–EEG responses observed in the present work can be used to characterise the subset of neurons in the pre–SMA involved in diverse behaviour. This will have importance in the context of physiological studies, especially involving motor planning, and in clinical studies in which the pre–SMA has received attention as a potential therapeutic target with repetitive TMS [68].

## Figures and Tables

**Table 1:** E–field values for the 12 conditions explored (measured in V/m).

RMT	0°	90°	180°	270°
100% RMT	110	108.8	90.5	90.6
120% RMT	132.2	130.9	108.8	108.9
140% RMT	154.4	152.9	127.1	127.2

**Table 2:** Summary statistics of the ANOVAs on TEP peaks.

	CO		SI		CO × SI	
	F_3,45_	*p*	F_2,30_	*p*	F_6,90_	*p*
PI	6.763	0.001	1.031	0.369	0.289	0.941
PII	1.709	0.179	2.646	0.087	3.520	0.004
PIII	7.697	<0.001	0.475	0.626	2.355	0.037
PIV	4.131	0.011	8.899	0.001	0.283	0.944
PV	7.316	<0.001	4.504	0.019	0.743	0.617

## Data Availability

Data are available upon request to the corresponding author.
